# Comparison of machine learning classification and regression models for prediction of academic performance among postgraduate public health students

**DOI:** 10.1038/s41598-025-31023-z

**Published:** 2025-12-18

**Authors:** Amira Fathy Abdallah Sayed, Mostafa Ahmed Arafa, Nessrin Ahmed El-Nimr, Karim Ahmed Samy Banawan, Marwa Shawky Abdou

**Affiliations:** 1 Department of Epidemiology, High Institute of Public Health Alexandria University, Alexandria, Egypt; 2 Borg El Arab Central Hospital (Baheeg clinics), Ministry of Health and population, Alexandria, Egypt; 3 Department of Electrical Engineering Faculty of Engineering, Alexandria University, Alexandria, Egypt; 4 Electronics and Communications Engineering Department, American University in Cairo, Cairo, Egypt

**Keywords:** Academic performance, Postgraduate public health, Prediction, Machine learning, Medical research, Mathematics and computing

## Abstract

Machine learning (ML) is an artificial intelligence tool that focuses on learning by generating models using established algorithms that represent a given dataset. It can be used as a predictive tool for students’ academic performance (AP) at both undergraduate and postgraduate levels. A cross-sectional analysis was conducted using academic records of 922 postgraduate students admitted to the High Institute of Public Health, Alexandria University, Egypt, between 2020–2024. Data included 22 features spanning pre-enrollment metrics, academic performance, and demographic traits. Classification algorithms, and regression models were trained on 75% of the dataset, validated via 5-fold cross-validation. Performance metrics included accuracy, precision, recall, AUC for classification, and MAE, RMSE, and R² for regression. Regression models outperformed classification models in AP prediction, with Ensemble (Soft Voting) achieving the highest accuracy (74.25%), lowest MAE (0.3383), and RMSE (0.4316). Among classification models, Random Forest demonstrated superior accuracy (71.43%) and AUC (0.87). Numerical features like the number of failed courses showed the strongest negative correlation with AP (*r* = -0.37). Key predictors included bachelor’s university, major, department, and pre-enrollment CGPA. Feature importance analysis highlighted failed courses as the top determinant, followed by institutional and academic background variables. Regression-based ML models, particularly Ensemble (Soft Voting), proved more effective than classification approaches for predicting nuanced variations in AP. These findings enable institutions to prioritize early interventions for at-risk students, and optimize resource allocation. However, moderate R² values (0.3832) underscore the need to integrate psychosocial and behavioral factors in future studies.

## Introduction

Public health (PH) is a priority in all societies worldwide. It is a branch of healthcare concerned with preserving and enhancing the health of the population. A key element of any effective and scientifically planned health policy in any country is being a competent PH specialist. The PH candidate selection process is an essential element to ensure this competency. Admission criteria differ between PH schools and programs; however, a common factor among them is the grade point average (GPA) of the bachelor’s degree, which has been shown to be a predictor of success, as measured by the cumulative GPA (CGPA) of PH master’s students at the Medical University of Warsaw^1^. On the other hand, adopting a holistic approach that includes aspects beyond quantitative measures, such as demographic characteristics and academic background, as predictors of student outcomes can help select successful students who will eventually contribute their skills to the PH workforce^2^.

In general, the educational process has many aspects. One of them is academic performance (AP), which refers to a student’s grades (academic achievement), the skills and knowledge they develop, how much they have learned, the career they eventually pursue, and their perseverance in their studies^3,4^. AP can be measured and predicted by assessing various factors that influence students using integrated techniques and methodologies^3^. Many factors have been shown to predict AP, including sociodemographic characteristics, online learning, academic management, and psychosocial and academic environments^3,5^. The diversity of these factors, together with the availability of digital data regarding the entire learning process across different institutions, opens the door to the use of modern techniques such as learning analytics (LA) and educational data mining (EDM) for data processing and performance prediction^6^. Machine learning (ML) focuses on learning by generating models using established algorithms that represent a given dataset. More simply, it investigates the ability of computers to learn or improve their performance based on data^7,8^. It is one of the most important approaches to modeling, along with statistical modeling^9^. Four learning methods are commonly used under the umbrella of ML: supervised, unsupervised, semi-supervised, and reinforcement learning^7^.

Predicting AP of PH students helps institutions identify high-performing and struggling learners, as well as their strengths and weaknesses. This enables the targeted allocation of resources (e.g., scholarships and development funds) to reduce attrition-related waste and improve outcomes through timely educational adjustments. Ultimately, this fosters a skilled public health workforce, indirectly enhancing the impact^10,11^. To the best of our knowledge, this is the first study conducted in the Middle East, aiming to develop reliable and accurate ML algorithms for predicting AP among postgraduate PH students and to identify key correlates influencing academic outcomes, especially since the High Institute of Public Health (HIPH) is the only one in Egypt and Arab countries that grants master’s and doctoral degrees for postgraduate public health students.

## Materials and methods

### Study setting and design

This cross-sectional study was conducted at the HIPH, Alexandria University, Egypt, between July and December 2024. The first postgraduate institute in the Eastern Mediterranean region specializes in public health.

### Target population and sampling

A total of 922 academic and administrative records of postgraduate PH students admitted for a PH degree between 2020 and 2024 (four academic years) who had completed at least two semesters were included in the study.

### Data collection tool

Data were collected using an academic and administrative records review sheet consisting of 22 factors (features). These features included the following: socio-demographic and personal characteristics: age, gender, nationality (Egyptian,1 & Non-Egyptians 2), marital status, current job, and residence), pre-enrollment characteristics (university of graduation, faculty, specialty, cumulative grade point average (CGPA), and English test score), and academic data (specialty, program, semester of registration, number of complementary courses (if applicable), average number of courses registered per semester, number of registered courses, number of failed, withdrawal, or forced withdrawal courses, number of credit hours registered (courses only), and CGPA.

**Fig. 1:**
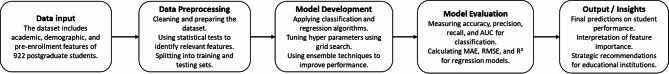
Data workflow.

The target variable is students’ final CGPA (used as the indicator of AP), treated both as: A continuous variable (for regression, scale 0.0–4.0), and A 3-class categorical variable (for classification, using institutional thresholds): high and very high graduate caliber: 3.3–4.0, satisfactory performance: 2.0–3.2, and less than expected and unsatisfactory, or fail: 0.0–1.9. The stratification reflects institutional grading policy at HIPH, Alexandria University, where these categories correspond to official academic standing, which mirrors real-world decision thresholds used by academic committees for probation, scholarships, or warnings, Fig. 1.

### Data exploration

The dataset was split into training (75%) and testing (25%) sets using stratified sampling to ensure that the proportion of students in each CGPA category (high, satisfactory, unsatisfactory) remained consistent across both subsets, Fig. 1.

### Feature engineering

The Chi-square independence test was used on the categorical features of the original dataset to determine which features related to the target value (CGPA Grade) the most. For numerical data, Pearson’s correlation was calculated for all features with the target value, Fig. 1.

### Modeling

All models were trained using scikit-learn version 1.4.1^12^. with default or grid-search-optimized hyperparameters; a fixed random seed (42) ensured reproducibility across runs. This process consists of testing different approaches as well as multiple models with various hyperparameters, comparing their performance on each dataset, and then selecting a few to fine-tune and obtain the best results. The ensemble learning technique used in every approach consisted of combining different trained models into one algorithm. In our case, the top five performing models were chosen. Hyperparameters for all models were fine-tuned using the grid search method, and 5-fold cross-validation was utilized to evaluate model stability, generalizability, and overfitting risk, with performance metrics averaged across folds to ensure robustness. The grid parameters varied across the different models. Additionally, a consistent random seed was used to ensure identical training and validation sets across the various ML algorithms, Fig. 1.

### Evaluating the model

To measure and evaluate the algorithms’ quality, four evaluation methods were used for the classification algorithm: **accuracy** [the ratio between the accurately classified values (true negatives and true positives) and the total dataset], **precision (confidence or true positive accuracy)** [is the measure of accuracy of predicted positives; the proportion of the predicted positive cases that are really positive (true positive) from all predicted positive values (true positive + false positive)], **recall (sensitivity or true positive rate) [**is the fraction of the real positive cases (true positives) that were accurately predicted as positive from all positive values (true positives + false negatives)], and **Area Under the Curve (AUC) [**is the measure of the area under the Receiver Operating Characteristic (ROC) curve, which plots the True Positive Rate (TPR) against the False Positive Rate (FPR) at various classification thresholds]^13^. F1 and Mathhews correlation coefficient (MCC) were applied in case if imbalanced data. For the regression algorithms, we used the mean absolute error (MAE), root mean square error (RMSE), and R square as evaluation measures^14^.

We selected widely tested and interpretable models (e.g., Random Forest, Logistic Regression, kNN, and Ensemble methods) because they offer a balance between predictive accuracy and interpretability, which is essential for educational decision-making. Additionally, these models provide robust baselines against which more complex approaches can be compared, Fig. 1.

## Results

The distribution of socio-demographic and academic features of the data set shows that the majority of the students lay in the age group 20–29 and 30–39, both representing 42.9% of the sample. Females represented 78.5%, and there was a nearly equal distribution between single (48.8%) and married (47.6%) students. The majority were Egyptians (94.9%), worked (81.4%), and had graduated from governmental universities (82%). About 57% of the students were enrolled in diploma programs, 33.1% in academic master programs, 1.5% in professional master programs, and 8.3% in doctorate programs. Half of the students had a “very high graduate caliber” CGPA (54.8%), 32.9% had a “satisfactory performance” CGPA, and 12.3% had “less than expected, unsatisfactory or fail”.

Table 1 shows the results of Pearson’s correlation test for numerical features with students’ CGPA. The feature with the highest correlation with the target value was the number of F courses, where *r*= −0.37.

**Table 1:** Pearson’s correlation test results for features of the postgraduate PH students with the target outcome (Alexandria, 2024).

Features	*r*	*P* value
Registration age	0.079	0.78
No. of complementary courses	0.11	0.66
No. of registered courses	0.023	0.3
No. of Fail (F) courses	−0.37	0.001*
No. of Withdrawal (W) courses	−0.16	0.12
No. of Forced Withdrawal (FW) courses	−0.25	0.23
No. of Credit Hours (CH) registered	0.12	0.33
Average no. of registered courses each Semester	0.042	0.5

*p-value ≤ 0.05.

Table 2 shows a comparison between the different classification algorithms tested in terms of the evaluation metrics calculated for each algorithm. Random forest (RF) was the best classifier algorithm for the target value in terms of accuracy (71.43%), precision (0.81), recall (0.53), and AUC (0.87), followed by ensemble with an AUC of 0.86, and logistic regression with an AUC of 0.86. Random Forest also remains the top-performing classifier in terms of (F1 = 0.68, MCC = 0.52), reinforcing our original conclusion while offering a more nuanced evaluation.

**Table 2:** Performance indicators for classification algorithms used in predicting AP of postgraduate PH students (Alexandria, 2024).

Classification algorithms	Original Dataset					
	Accuracy %	Precision	Recall	micro average AUC	F1	MCC
Random Forest	**71.43**	**0.81**	**0.53**	**0.87**	**0.68**	**0.52**
XGB	66.67	0.77	0.50	-	0.52	0.40
Linear SVC	66.23	0.77	0.49	-	0.60	0.39
CatBoost	64.07	0.75	0.49	-	0.59	0.37
kNN	67.1	0.78	0.51	0.86	0.61	0.42
Poly SVC	65.8	0.60	0.49	-	0.54	0.38
RBF	67.53	0.45	0.46	0.86	0.45	0.40
Logistic Regression	68.4	0.79	0.51	0.86	0.61	0.44
Ensemble	70.13	0.80	0.52	-	0.63	0.50

Table 3 shows a comparison between the different regression algorithms tested in terms of the evaluation metrics calculated for each algorithm. The Ensemble (Soft Voting) was the best regression algorithm for the target value in terms of accuracy (74.25%), followed by kNN regression with an accuracy of 73.39%, and RBF SVR regression with an accuracy of 69.96. In addition, Ensemble (Soft Voting) showed the least MAE and RMSE values and the highest R^2^ value among all tested regression algorithms, indicating that Ensemble (Soft Voting) on average had an absolute difference between its predictions and the actual values of 0.3383 units, and its predictions were about 0.4316 units away from the actual values, indicating the presence of a few outliers in the dataset. However, its R^2^ was low as it explained approximately 38% of the variance in the target variable.

**Table 3:** Performance indicators for regression algorithms used in predicting AP of postgraduate PH students (Alexandria, 2024).

Regression algorithms	Accuracy %	MAE	RMSE	*R* ^2^
ElasticNet regression	65.67	0.3694	0.4662	0.28
Ridge regression	66.95	0.366	0.462	0.2932
Random Forest regression	68.24	0.364	0.4601	0.2992
XGB Regressor	65.24	0.3661	0.4978	0.1796
Linear SVR regression	66.95	0.3662	0.4619	0.2935
kNN regression	**73.39**	**0.3437**	**0.4456**	**0.3424**
Poly SVR regression	66.95	0.3502	0.4557	0.3124
RBF SVR regression	69.96	0.3675	0.4879	0.2119
Ensemble (Soft Voting)	**74.25**	**0.3383**	**0.4316**	**0.3832**

**Fig. 2:**
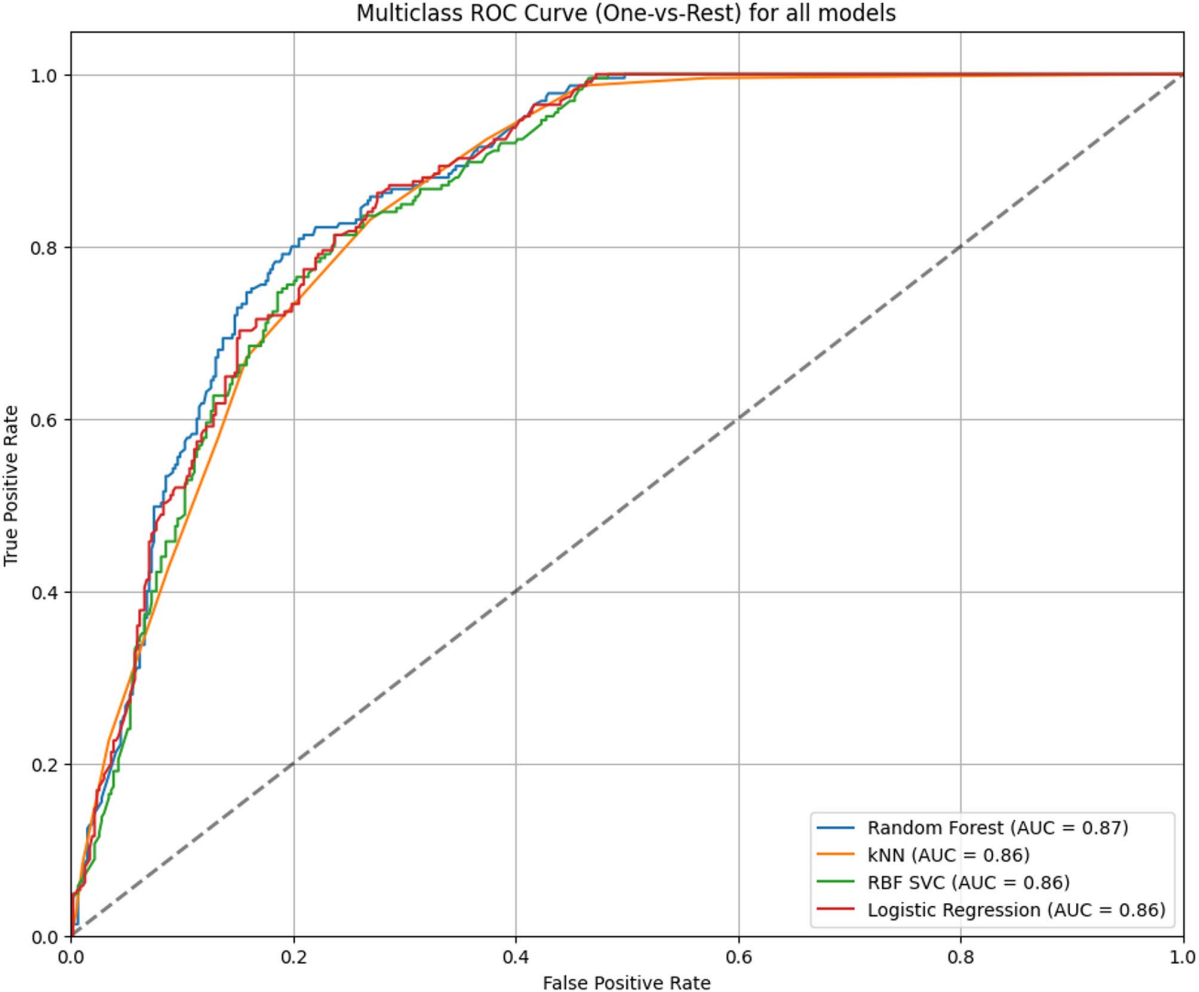
Micro average AUC for the top 4 performing algorithms used in predicting AP of postgraduate PH students (Alexandria, 2024).

Figure 2 shows the micro-average AUC for the top-performing classifiers. RF showed the highest AUC of 0.87, which indicated a very good ability to distinguish between the three classes of CGPA, followed by kNN, LR, and RBF with an equal AUC of 0.86, which also indicated a very good classification ability of these classifiers.

**Fig. 3:**
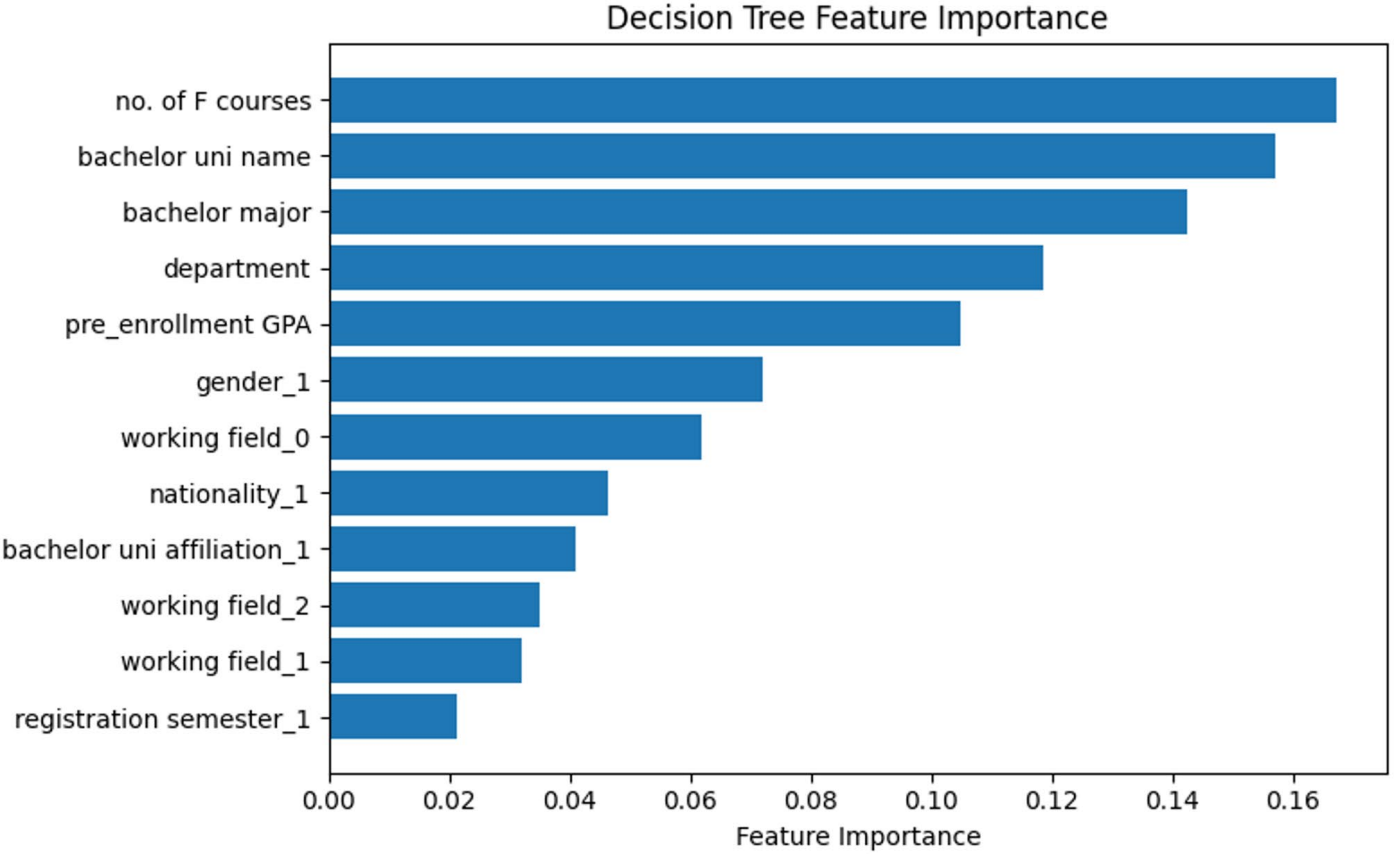
Decision tree feature importance of postgraduate PH students’ dataset (classification).

Figure 3 shows the relative importance of different features, as determined by a decision tree model using classification algorithms. “no. of F courses” was the most important feature that correlated with prediction, followed by the university from which the bachelor’s degree was obtained and bachelor’s major. Department and pre-enrollment GPA ranked 4th and 5th, respectively. Others such as registration semester, working field, and bachelor university affiliation had less impact on the classification model’s decisions.

**Fig. 4:**
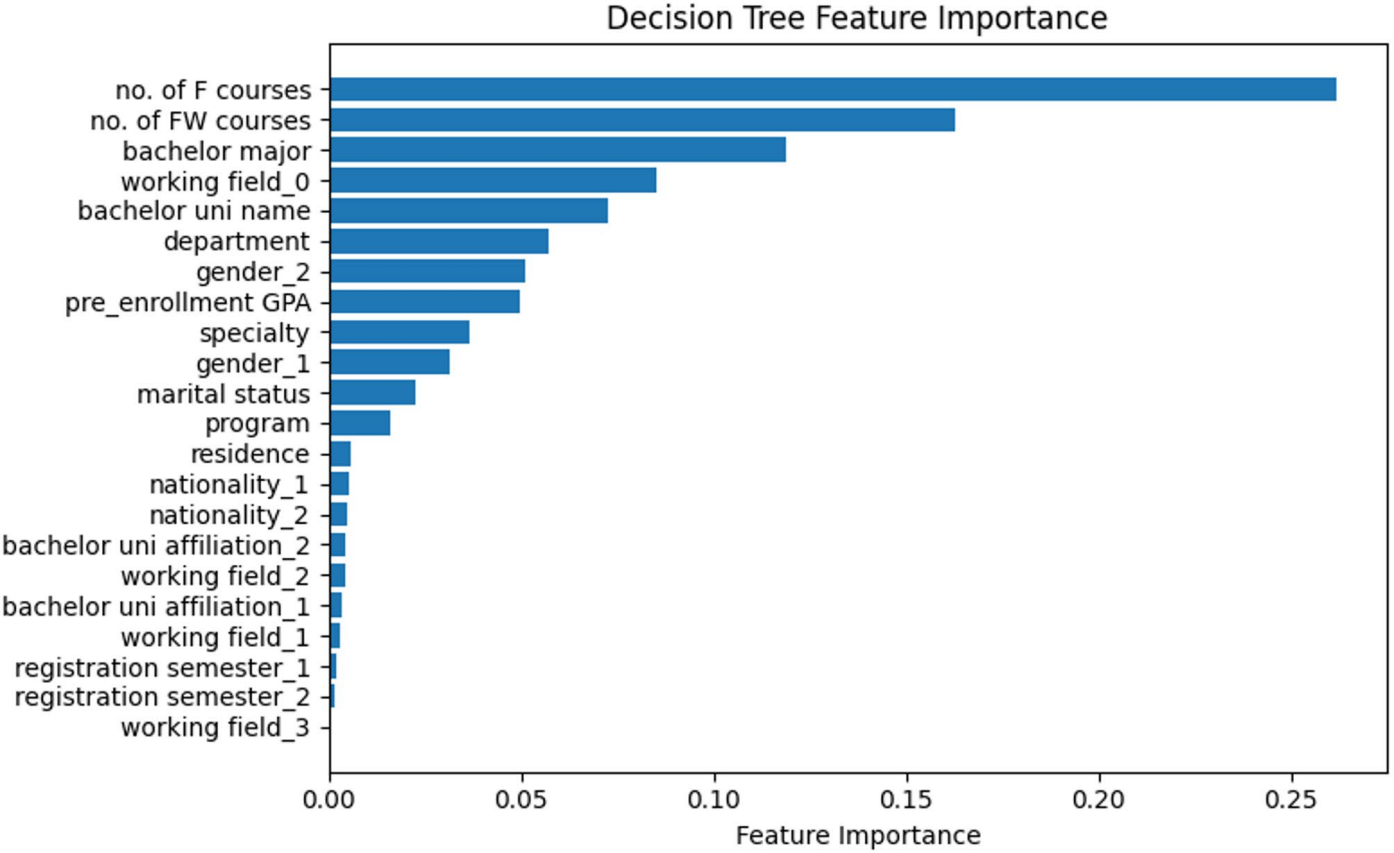
Decision tree feature importance of postgraduate PH students’ dataset (regression).

Figure 4 shows the relative importance of different features, as determined by a decision tree model using regression algorithms. Number of F courses was the most important feature, followed “number of forced withdrawal courses’, and bachelor major ranking 2nd and 3rd respectively. Others, such as registration semester, working field, bachelor’s university affiliation, nationality, and residence, had less impact on the regression model’s decisions.

**Fig. 5:**
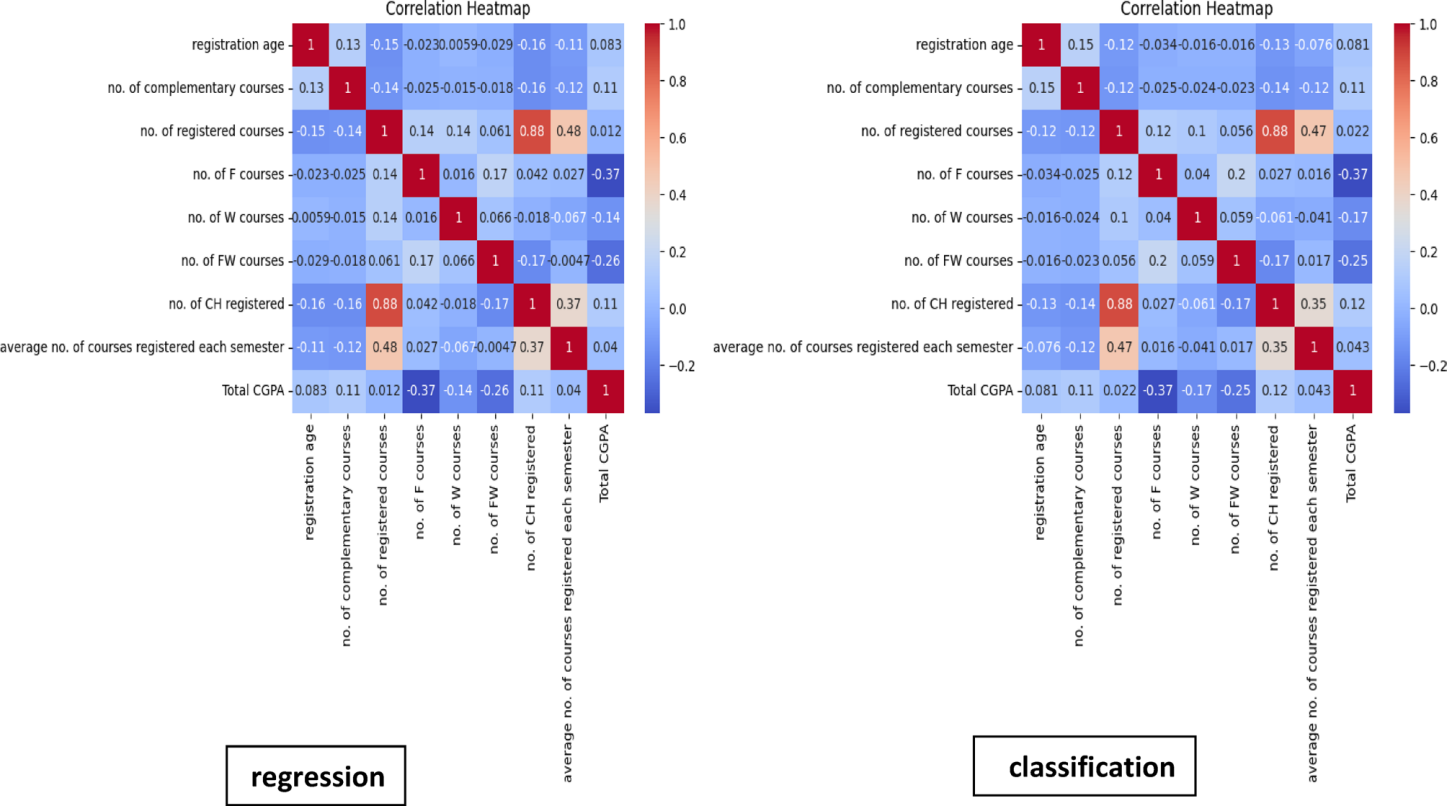
Heat map correlation matrix for features used in predicting AP of postgraduate PH students (Alexandria, 2024).

Figure 5 shows the linear relationships between features and CGPA and between features and each other regarding the classification models. This shows that the final CGPA has a strong negative linear relationship with “no. of F courses, " −0.37; a moderate negative relationship with “no. of FW courses, " −0.25, a weak negative relationship with “no. of W courses” −0.17, “no. of complementary courses’ 0.11, and “no. of CH registered” 0.12, a relatively weak or no relationship with age 0.081, and “average no. of registered courses’ 0.043. while " no. of registered courses” showed strong positive correlation with " no. for CH registered” 0.88, and a weak positive correlation with “average no. of registered courses’ 0.47. In contrast, the other features showed weak negative or near-zero correlations. regarding the regression models. They showed the same direction and strength of linear correlations between features as those shown in the classification models.

## Discussion

This study compared machine learning classification and regression algorithms for predicting the academic performance of postgraduate PH students. Classification and regression models serve different analytical purposes. Classification categorizes students into performance groups (e.g., high, satisfactory, unsatisfactory), enabling rapid screening and intervention planning. Regression, on the other hand, predicts continuous CGPA values, offering finer granularity for academic forecasting and resource optimization. This dual modeling approach aligns with established practices in educational data mining and allows institutions to choose the method that best fits their operational needs. Regression models, particularly the Ensemble (Soft Voting) approach, outperformed classification algorithms, achieving 74.25% accuracy compared to the best classification model (Random Forest, 71.43% accuracy). This aligns with the findings of Ying and Ma 2024 in China, who demonstrated that regression approaches surpass classification in predicting multi-level student performance^15^, and Tulawe et al. 2023 in the Philippines, who found better performance for regression trees over classification trees^16^, owing to the regression ability to capture continuous variations in CGPA. Classification simplifies outcomes into discrete categories (e.g., “very high,” “satisfactory”). Regression inherently accounts for nuanced differences in academic scores, reducing information loss, and improving predictive power.

Similarly, Yohannes and Adem (2018), in their work on 134 undergraduate students in Ethiopia, found that regression models (LR and SVR) performed better than the classification model (Neural Networks) in predicting students’ AP^17^. However, another study conducted in 2015 observed better results for classification models than regression models obtained from their analysis of students in Porto, Portugal^18^.

Notably, the Random Forest (RF) classifier achieved the highest AUC (0.87), indicating strong class separation, which indicates an 87% chance that the RF model will define which student belongs to which CGPA class correctly. In our data, where the “fail” category represents only 12.3% of students, reporting F1-macro and MCC would provide a more honest assessment of whether our models can actually identify students who need intervention. These metrics align with best practices in educational data mining where identifying at-risk students is critical, even when they represent a small proportion of the overall student population^19^. However, the regression’s higher accuracy and lower error metrics (MAE: 0.338, RMSE: 0.432) suggest that it is better suited for precise CGPA predictions. Since CGPA is on a 4.0 scale, an MAE of 0.338 means the model’s predictions are, on average, off by ~ 0.34 grade points e.g., predicting 3.0 when the true value is 3.34. This is substantial in academic terms (e.g., B vs. B+), but acceptable for early-warning systems. RMSE = 0.432 further indicates the presence of some larger errors (outliers), consistent with educational data variability. These results underscored the regression as being more robust for AP prediction in contexts with granular outcome data. This supported by empirical review of Agyemang et al. 2024, who concluded that RF is the best classifier model for predicting AP^20^, and Alshanqiti & Namoun who emphasized using hybrid regression models and its superiority in predicting AP^21^. The results of the current study were comparable to the findings of other studies, which reported that the RF classifier emerged as one of the best models, achieving high accuracies of 78.3%, 92.60%, and 83.71%^22–24^. Whereas Logistic regression (LR) showed an accuracy of 68.4% in the current study, a higher percentage (85%) was reported by Wang et al. 2022 in China, while Ojajuni et al. 2021 in the USA reported a much lower percentage of accuracy for LR (40.96%)^23,24^. Among the regression algorithms, we found that Ensemble (Soft Voting) was the best in terms of accuracy (74.25%), MAE (0.3383), RMSE (0.4316), and R^2^ (0.3832), which was in accordance with the results of Amrieh et al. (2016) in Jordan, and in the literature review of Chen et al. 2025^25,26^. Generally, Ensemble techniques enhance model accuracy by reducing overfitting, improving generalization, and reducing variance and bias. The moderate R² value (0.38) indicates that while the model explains a significant portion of the variation in students’ CGPA, there remains a substantial amount of unexplained variance, largely attributed to the absence of psychosocial, behavioral, and contextual factors known to influence academic outcomes. Nevertheless, the relatively low MAE and RMSE of the model suggest that it is sufficiently accurate for practical applications, such as early intervention planning and resource allocation. Several studies in the field have reported similar or even lower R² values when predicting academic performance using machine learning models. For example, Wang and Luo (2024) noted that interpretable regression models in educational contexts rarely exceed an R² of 0.5, without incorporating real-time behavioral data^27^. Both classification and regression approaches serve distinct purposes in educational analytics. Classification and regression models serve different analytical purposes.

Key features influencing AP were consistent across classification and regression models, although their relative importance varied. The number of failed courses (F courses) emerged as the strongest predictor (Pearson’s *r* = −0.37), reflecting its critical role in academic progression. This aligns with the findings of Ojajuni et al., who identified prior academic failure as a dominant predictor of AP in undergraduate settings^23^. The strong negative correlation between failed courses and CGPA suggests that early identification of students at risk of failing even one course could significantly improve retention and graduation rates. Institutions may consider targeted interventions such as remedial tutoring or personalized learning plans starting from the first semester.

Other significant correlates included bachelor’s institution and major: governmental universities and specific majors (e.g., public health-aligned disciplines) correlated with higher CGPA, consistent with Al-Alawi et al., who linked institutional prestige and field-specific alignment to academic success^22^. Pre-enrollment CGPA demonstrated moderate predictive value, supporting Alyahyan and Düştegör’s meta-analysis on pre-admission metrics in 2020^5^. Among the demographic factors, gender and nationality showed weaker associations, corroborating other studies that found no significant gender-based AP differences between undergraduate and postgraduate cohorts^23,28^.

Regression-specific features like “specialty” and “FW courses” (forced withdrawn courses) gained prominence, likely due to regression’s sensitivity to continuous relationships. This highlights the need for tailored feature selection based on the modeling approach, as emphasized by Wang and Luo 2024 in China^27^.

While predictive models can support early interventions and resource allocation, they should never replace holistic, human-in-the-loop decision-making, especially in high-stakes contexts like admissions. Individual circumstances, resilience, and contextual factors beyond quantifiable metrics must always be considered to ensure fairness and equity in education.

### Limitations

While regression models outperformed classification models, both approaches were constrained by dataset imbalances and limited feature diversity (e.g., psychosocial variables). Despite the relatively small sample size, it represented the vast majority of postgraduate PH students in Egypt during this given period. Lastly, our dataset was limited to the single institution in Egypt and the Arab world. Therefore, while our findings are significant within this context, caution should be exercised when generalizing to other settings without further validation.

## Conclusions

This study addresses a critical gap in regional educational research and provides actionable insights to inform targeted interventions and resource allocation in higher public health education institutions. This study compared machine learning classification and regression models to predict AP among postgraduate public health students. The analysis revealed that regression models, particularly the Ensemble (Soft Voting) approach, outperformed classification algorithms, achieving 74.25% accuracy, while the RF classifier emerged as the top-performing classification model with 71.43% accuracy. Key predictors of AP included the number of failed courses, bachelor’s institution, department, and pre-enrollment CGPA. The study findings advocate for regression-based models in higher education AP prediction, particularly for resource allocation (e.g., scholarships and remedial programs). However, a moderate R² underscores the complexity of AP, which is influenced by unmeasured psychosocial or environmental factors. Future work should incorporate larger, multi-institutional datasets and hybrid models (e.g., deep learning) to address nonlinear relationships, as recommended by Alshanqiti & Namoun, and Wang et al.^21,24^.

## Data Availability

All data and material are presented within the manuscript.

## Abbreviations

AP: Academic performance
AUC: Area Under the Curve
CGPA: Cumulative grade point average
EDM: Educational data mining
FPR: False Positive Rate
GPA: Grade point average
HIPH: High Institute of Public Health
LA: Learning analytics
MAE: Mean absolute error
ML: Machine learning
RMSE: Root mean square error
PH: Public Health
ROC: The Receiver Operating Characteristic
TPR: True Positive Rate
